# Review of Clinical Studies of the Treatment of Ulcerative Colitis Using Acupuncture and Moxibustion

**DOI:** 10.1155/2016/9248589

**Published:** 2016-11-03

**Authors:** Jun Ji, Yan Huang, Xia-Fei Wang, Zhe Ma, Huan-Gan Wu, Hyoyoung Im, Hui-Rong Liu, Lu-Yi Wu, Jing Li

**Affiliations:** ^1^Shanghai Research Institute of Acupuncture and Meridian, Shanghai University of Traditional Chinese Medicine, Shanghai 200030, China; ^2^Key Laboratory of Acupuncture-Moxibustion and Immunology, Shanghai University of Traditional Chinese Medicine, Shanghai 200030, China; ^3^Yueyang Clinical Medical College, Shanghai University of Traditional Chinese Medicine, Shanghai 200437, China; ^4^Department of Acupuncture and Moxibustion, Shanghai Yueyang Hospital of Integrated Traditional Chinese and Western Medicine, Shanghai University of Traditional Chinese Medicine, Shanghai 200437, China

## Abstract

*Background.* Clinical studies suggest that acupuncture and moxibustion therapy in ulcerative colitis (UC) can regulate bowel inflammation, and these treatments have the advantages of low rates of adverse reactions and recurrence as well as good long-term efficacy. We reviewed the current status of clinical studies of the treatment.* Methods.* Randomized controlled trials (RCTs) using the therapy as the major intervention for treating UC were included from 1995 to 2015. The extracted data mainly included diagnostic standards, treatment methods, selection of acupoints, treatment times and courses, and efficacy determination criteria.* Results.* The use of diagnostic standards and efficacy criteria lacked unification and standardization. There were two main groups: acupuncture and moxibustion therapy combined with drug treatment and the use of all types of acupuncture and moxibustion therapy alone or in combination. The acupoint compositions included distal-proximal point combinations, back-shu point and front-mu point combinations, and acupuncture through meridians. The treatment courses in all the clinical trials had large variations.* Conclusion.* The treatment of UC in the examined articles was mainly based on the classical theory. However, many links of the clinical regimen design were still lacking, which affected the repeatability of the clinical studies and the accuracy of the clinical conclusions.

## 1. Introduction

Ulcerative colitis (UC) is a chronic, nonspecific inflammatory disease with unknown etiology. UC mainly invades the distal colon, rectal mucosa, and submucosa, and it can involve the entire colon or even the terminal ileum. Its clinical manifestations are mainly recurrent mucopurulent bloody stool, abdominal pain, diarrhea, and combined systemic presentations to different degrees. The disease course is very long which can result in the formation of intestinal fibrosis and stenosis. The age of disease onset is mainly young adults aged 20–40 years. There is no difference between males and females. Generally, the disease has slow development and different severity and is prone to recurrence. Some disease courses can last for several years or even several decades. UC is closely associated with the development of colon cancer and is recognized by the World Health Organization (WHO) as a refractory bowel disease.

The pathogenic mechanism of UC is still not completely clear. Modern medicine considers the development of UC to be associated with a variety of factors, mainly including environmental factors, immune factors, inflammation, eating disorders, emotional distress, and genetics. Currently, there is no radical therapy for UC. UC is mainly treated with aminosalicylic acid preparations {salazosulfapyridine (*SASP*) and aminosalicylic acid (ASA)}, steroid hormones, immunosuppressants, and new biologics. The goal of treatment is to maximally relieve symptoms, eliminate inflammation, heal ulcers, prevent complications, and prevent recurrence. However, the efficacy of treatment using the above drugs has large individual differences, and there are different levels of toxic side effects.

Based on its clinical manifestations, this disease can be classified into the scope of “intestinal afflux,” “diarrhea,” “chronic diarrhea,” “chronic dysentery,” and “bloody stool” in traditional Chinese medicine. Symptoms are mainly caused by feeling pathogenic dampness, improper diet, emotional disorders, and weakness of the viscera. Therefore, the disease location is mainly the large intestine and is closely associated with the spleen and stomach; in addition, UC usually involves the liver and kidney. Currently, the treatment of this disease using traditional Chinese medicine mainly involves therapeutic methods such as the administration of traditional Chinese medicines orally or via enemas, acupuncture, and moxibustion. Since the 1990s, clinical studies on the treatment of UC using acupuncture and moxibustion have gradually increased. Current studies show that acupuncture and moxibustion can regulate physiological balance at multiple links and multiple targets in the body to effectively control bowel inflammation. In addition, these treatments have the advantages of low adverse reactions, low recurrence, and good long-term efficacy. Therefore, these treatments have been applied extensively.

A review of clinical studies in this field based on randomized controlled trials (RCTs) of UC treatment using acupuncture and moxibustion published between 1999 and 2015 was performed, and the results are described below.

## 2. Materials and Methods

### 2.1. Retrieval Strategy

The searched databases included foreign language databases and Chinese databases. Foreign literature on registered RCTs was searched in Medline, EMBASE, and the Cochrane Library. The search was limited to human studies and RCTs. Chinese literature was searched in the following databases: the China National Knowledge Infrastructure Database (CNKI), the Chongqing VIP Chinese Science and Technology Periodical Database (VIP), and the Chinese Biomedical Literature Database (CBM). The search period was from January 1, 1995, to December 31, 2015. The Chinese Medical Subject Headings (MeSH) terms included “inflammatory bowel disease”, “ulcerative colitis”, “acupuncture”, “moxibustion”, “acupoint”, and “acupuncture and moxibustion therapy”. The English MeSH terms were “acupuncture”, “moxibustion”, “inflammatory bowel disease”, and “ulcerative colitis”. According to the specific conditions of the different databases, a comprehensive retrieval using MeSH terms combined with key words and free words was performed to ensure the completeness of the search results.

### 2.2. Inclusion Criteria

(1) Study subjects: patients with a definitive diagnosis of UC were included. There were no restrictions on race, age, or gender. (2) Study design: RCTs of UC treatment using acupuncture and moxibustion were included. The languages were limited to Chinese and English. (3) Intervention measures of experimental groups: experimental groups mainly received acupuncture and moxibustion therapy (including filiform needle acupuncture, electroacupuncture, moxibustion, and cupping therapy) alone or combined with other treatment methods (such as drug therapy). In addition, needling methods, acupoint selection, and needle material were not further classified. For acupuncture and moxibustion combined with drug therapy, the drugs administered to experimental groups and control groups in the same study should be consistent. (4) There were no restrictions on the intervention measures used in control groups. (5) Literature with full articles or abstracts that provided sufficient information was included.

### 2.3. Exclusion Criteria

Literature was excluded due to the following conditions: (1) study subjects and intervention measures that did not conform to the inclusion criteria; (2) RCTs without clear diagnostic standards, basic information on subjects, or intervention measure-related information; (3) series of observations, case reports, expert experiences, and descriptive analyses without controlled cases; and (4) literature with duplicated detection or duplicated publication.

### 2.4. Data Extraction

Two researchers independently performed literature screening, modified Jadad quality scoring, data information extraction, and database establishment. A third assessor checked the data for consistency. Cases of inconsistent data were resolved by discussion. The extracted data mainly included the number of published studies, diagnostic standards, treatment methods and results, acupoint and meridian selection, treatment times and courses, and efficacy determination criteria.

## 3. Results

**Figure 1:**
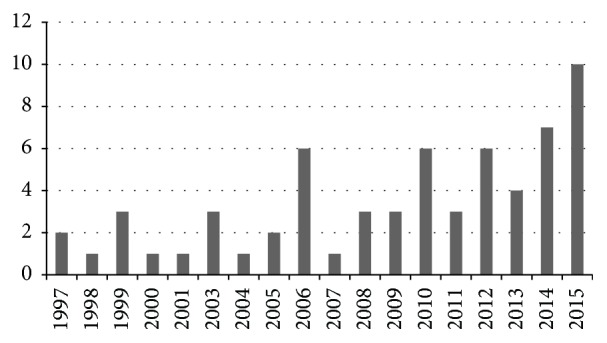
Number of clinical study articles.

According to the retrieval strategy, a total of 872 Chinese and English literature reports were retrieved from the above medical databases. A bibliography of the literature was introduced into Excel, and repeated bibliographies were deleted. After reading titles and abstracts, significantly unrelated articles were excluded. The full articles were downloaded and read. Unrelated articles were deleted according to the study's literature exclusion criteria. Finally, 63 articles were included [1–63]. These articles included 58 Chinese articles and 5 English articles. The literature types included journal articles and graduate student degree dissertations. The number of clinical study articles on this disease was stable every year. The lowest annual number was 0 (2002), and the highest number was 10 (2015). Overall, the numbers showed a rising trend (Figure 1).

### 3.1. Diagnostic Standards

Summary statistics was performed on the 63 included UC articles. The results showed that 58 of the articles described the diagnostic standards that were used, whereas 5 articles [25, 29, 30, 47] did not explain these standards. Among the diagnostic standards adopted by these 58 articles, consensus opinions formulated by Chinese industry associations or expert committees were used in 8 articles, the practical guidelines for the diagnosis and treatment of inflammatory bowel diseases of the World Gastroenterology Organization were used in 1 article, and the other articles used standards from different types of teaching materials and books. The two most frequently used diagnostic standards were the “1993 ulcerative colitis diagnostic criteria from the Taiyuan National conference on chronic noninfectious intestinal diseases” and the “2000 diagnostic standards from the Chengdu conference on inflammatory bowel disease of the Chinese Society of Gastroenterology, Chinese Medical Association.” For the past 2-3 years, the definitive diagnosis of UC has been divided into Western medicine diagnosis and traditional Chinese medicine diagnosis; the latter focuses on the confirmation of the classification of symptom differentiation. In the past, only one type of diagnostic standard was used; there were diverse types of standards, which were not restricted to conference consensuses. Moreover, domestically formulated standards were generally used; only 1 article used foreign standards [61] (Table 1).

### 3.2. Use of Acupuncture and Moxibustion Therapy

A statistical analysis of the intervention measures used in the experimental groups of the 63 included articles showed these measures were efficacy and mainly of two types. One type was acupuncture and moxibustion therapy combined with drug treatment. The drugs could be either Western medicines or traditional Chinese medicines. A small number of articles used combined Western and traditional Chinese medicines. The drug administration methods included oral, enema, and rectal administration. The other type of intervention measure was the use of acupuncture and moxibustion alone or in combination. The most commonly used acupuncture and moxibustion therapies included acupuncture, electroacupuncture, moxibustion, warm needling, acupoint catgut embedding, acupoint application, balance cupping, and auricular point sticking. The application of acupuncture combined with moxibustion was the most common intervention measure (Table 2). The use of moxibustion alone had the highest frequency, of which partitioned moxibustion was the most commonly used (Table 3).

### 3.3. Selection of Meridians and Acupoints

A statistical analysis of the frequency of acupoints used in acupuncture and moxibustion therapy in the experimental groups in the 63 included articles revealed the use of 55 acupoints in total. More than 10 articles used 12 acupoints, and the Tianshu (ST25) acupoint had the highest frequency of use (Table 4).

A statistical analysis of the meridian tropism, division, and specific point properties of all 55 acupoints showed that the clinical selection of acupoints for the treatment of UC using acupuncture and moxibustion involved a set of 10 regular meridians and 2 sets of an additional 8 extra-meridians. The most commonly used meridians were Yangming Stomach Channel of Foot, Taiyin Spleen Channel of Foot, Taiyang Bladder Channel of Foot, and Ren Meridian (Table 5).

The divisions mainly included the abdomen, back, and lower limbs. The utilization of specific points was as high as 69%. The major points were five-shu point, back-shu point, lower He-Sea point, mu point, and convergent point. The composition of acupoints exhibited the following features: (1) distal-proximal point combinations involving the combined use of abdominal acupoints (proximal) and lower extremity acupoints (distal); (2) back-shu point and front-mu point combinations, such as the combined use of Tianshu (ST25) and Dachangshu (BL25), Zhongwan (RN12) and Weishu, and Guanyuan (RN4) and Xiaochangshu; and (3) acupuncture through meridians. In addition to the commonly used acupoints, the corresponding meridian points were used in combination mainly based on symptoms or the differentiation of symptoms.

Among the 63 included articles, 52 articles described the treatment frequency, the number of treatments in each treatment course, and the total number of treatment courses. There were 46 articles describing the use of acupuncture and moxibustion intervention measures in experimental groups once daily. The most common treatment course comprised 10 treatments. The number of total treatments varied from 10 days to more than 70 days. A statistical analysis using 10 days as one segment showed that the total treatment time was most often 30 days or 10–19 days. The duration of each acupuncture and moxa stick moxibustion treatment ranged from 15 to 40 min; a treatment time of 30 min was the most common. Indirect moxibustion was generally applied for 3 Zhuangs. Some articles also reported 4–7 Zhuangs of moxibustion (Tables 6 and 7).

### 3.4. Efficacy Criteria and Outcome Indicators

The efficacy criteria and outcome indicators of the 63 included articles were statistically analyzed. Most articles used the efficacy criteria of the “1993 Taiyuan ulcerative colitis diagnostic criteria from the national conference on chronic noninfectious intestinal diseases,” the “principles of guidelines for clinical studies of new drugs in traditional Chinese medicine,” and self-formulated efficacy criteria (Table 8).

The outcome indicators included the total effectiveness rate, colon activity index, clinical symptom scores, fiber colonoscopy, laboratory indicators (including T lymphocyte subpopulations; immunoglobulins; tumor necrosis factor *α* (TNF *α*); interleukin- (IL-) 6, 8, and 10; and C-reactive protein), and adverse reactions to treatment measures. All 63 articles used the effectiveness rate as an outcome indicator. In addition, 10 articles [10, 29, 31, 40, 45, 47, 56–58, 61] used fiber colonoscopy as an outcome indicator, 16 articles used laboratory detection as an outcome indicator [3, 4, 6, 14, 15, 19, 28, 35, 38–40, 50, 57–59, 61], 3 articles used colon activity as an outcome indicator [44, 56, 57], 8 articles used symptom scores as an outcome indicator [10, 19, 21, 38, 44, 55, 57, 61], and 3 articles used intestinal mucosal pathology as an outcome indicator [8, 13, 36].

### 3.5. Clinical Trial Design

The included 63 articles contained a total of 5,404 cases of UC patients. There were 2,735 cases of male patients (50.6%) and 2,152 cases of female patients (39.8%). The gender of the other 517 patients in 7 articles was not reported. The average size of the trials was 87.8 cases; the smallest was 29 cases, and the largest was 640 cases.

An evaluation of the risk of bias showed that, among the 63 included RCT articles, 17 articles reported methods for the generation of random sequences [8–10, 16, 23, 27, 38, 39, 44, 46–49, 55, 56, 61, 63], and 3 articles used proper allocation concealment [8, 39, 44]. The methods of random allocation and allocation concealment of the other trials were not appropriate or were unclear. Four articles reported using blind methods [28, 35, 39, 44]; however, the descriptions of these methods in two articles [28, 35] were unclear. Two studies [39, 44] reported the numbers of cases removed from the trials and the reasons for removal. Two trials performed an estimation of sample size before the study [39, 44]. The quality of the methodology and reporting of the included articles was generally low. Six studies reported follow-up conditions [8, 20, 39, 51, 57, 61]. An evaluation of the quality of the 63 included articles using modified Jadad scoring showed that 3 articles [8, 39, 44] represented high-quality literature.

## 4. Discussion

### 4.1. Diagnostic Standards of Clinical Studies on UC Treatment Using Acupuncture and Moxibustion

The correct diagnostic standards are a prerequisite for the implementation of clinical studies. With continuous improvements in the in-depth understanding of UC in medicine, the diagnostic standards for this disease are also being constantly refined. From the diagnosis of clinical manifestations to the combination of lesion features under colonoscopy, the application of pathological examinations, endoscopic mucosal staining techniques, and magnification endoscopy are increasing the scientific rigor of diagnosis. In addition, disease condition assessment is an important component of UC diagnosis; this includes clinical type, lesion range, disease activity, severity, parenteral manifestation, and complications. These factors all have an important influence on the formulation of treatment regimens and the determination of clinical efficacy. However, this study revealed that the current diagnostic standards used for the treatment of UC with acupuncture and moxibustion exhibit a high degree of randomness. Most of the clinical trials adopted relatively old diagnostic standards, and even more studies used diagnostic standards described in different types of teaching materials and books. The variety of standards used had an influence on the accuracy of patient inclusion and thus affected the reliability of the studies' conclusions. In addition, the majority of articles did not report the disease assessment conditions of the included cases, which reduced the repeatability of the studies' conclusions and their reference value for clinical decision-making.

Currently, most consensus opinions and diagnostic standards rely on Western medicine methods because the features of the lesions of UC patients in China are similar to those in Western countries. The UC lesion range classification in the newest (2012) edition of consensus also recommends the 2008 European Crohn's and Colitis Organisation (ECCO) consensus and the 2010 Asian Pacific Association of Gastroenterology (APAGE) consensus [64]. However, physicians who practice acupuncture and moxibustion therapies guided by traditional Chinese medicine theories also should pay attention to syndrome differentiation-dependent treatment during disease differentiation-dependent treatment. Therefore, the diagnostic standards of symptom differentiation classification in traditional Chinese medicine should be considered. In the past 3 years, 9 articles [12, 38, 40, 44, 48, 49, 55, 58, 61] used the diagnostic standards of both traditional Chinese medicine and Western medicine, indicating that the diagnostic standards of traditional Chinese medicine have gradually received increasing attention. However, there are still fewer articles using the diagnostic standards of traditional Chinese medicine compared with Western medicine; the former needs attention from more researchers.

### 4.2. Acupuncture and Moxibustion Therapy Programs for UC

This study showed that the intervention measures applied to experimental groups in clinical trials of treatment for UC using acupuncture and moxibustion involved either acupuncture and moxibustion therapy combined with drugs or the use of acupuncture and moxibustion therapy alone or in combination. The results showed that the treatment effects in experimental groups were better than those in control groups. The selection of acupoints in trials mostly involved acupoints belonging to Yangming Stomach Channel of Foot, Taiyin Spleen Channel of Foot, Taiyang Bladder Channel of Foot, and Ren Meridian. Specific acupoints in the abdomen, back, and lower extremities of the above meridians were mainly used. These features were consistent with records in ancient literature [65, 66]. Currently, treatment regimens for UC using acupuncture and moxibustion have the following drawbacks. (1) Insufficient attention is paid to the combination of symptom differentiation acupoints. Only 2 articles [51, 54] formulated corresponding acupoint selection programs based on different symptom types. During the implementation of a RCT, the intervention measures used in the same group should remain consistent; in addition, the individuality of acupuncture and moxibustion treatments should be considered; therefore, to ensure the consistency of major treatment methods, treatments should be properly adjusted based on different symptom types of patients. (2) The setting of treatment courses is based on personal experiences, which leads to randomness. The results of this study showed that the examined clinical studies of the treatment of UC using acupuncture and moxibustion had large differences in needle retention time, the number of treatments in a single treatment course, the total number of treatment courses, and the number of Zhuangs in moxibustion treatment. The treatment course is associated with the properties of the disease and the intervention measures. In the future, studies examining the best treatment course for UC using acupuncture and moxibustion should be implemented to optimize the duration and interval time of treatment courses and to increase clinical efficacy. (3) The targets of the intervention measures of acupuncture and moxibustion are lacking. In UC, the lesion locations differ between the rectum and colon, the disease severities differ, and the attack period and remission period differ; therefore, the intervention measures of acupuncture and moxibustion should also differ accordingly. However, none of the 63 included articles described lesion locations, stages, or disease severity. Therefore, it was unclear whether the acupuncture and moxibustion intervention measures adopted in these studies are applicable to patients with different conditions.

### 4.3. Efficacy Criteria and Outcome Indicators of Clinical Studies on the Treatment of UC Using Acupuncture and Moxibustion

Since 1978, the Chinese Society of Gastroenterology of the Chinese Medical Association has issued 5 versions of UC diagnosis and treatment standards and efficacy assessment indicator systems. The existing efficacy assessment indicators have developed gradually in the industry and provide very good guidance for clinical applications [67]. In addition to self-formulation, most of the articles included in this study used the 2002 “principles of guidelines for clinical studies of new drugs in traditional Chinese medicine (provisional)” [68]. The UC efficacy assessment indicators proposed by this edition of principles include comprehensive efficacy, mucosal lesion efficacy, syndrome efficacy, major symptom efficacy, and related laboratory detection indicators; these indicators cover both traditional Chinese medicine and Western medicine and are comprehensive. However, the results of this study showed that the application of efficacy criteria in recent clinical studies of UC treatment using acupuncture and moxibustion was not sufficiently unified or standardized; most studies used old or self-formulated criteria. The inflammatory bowel disease study group of the Chinese Society of Gastroenterology of the Chinese Medical Association formulated the newest “consensus on the diagnosis and treatment of inflammatory bowel disease” in 2012 [69] to update and modify efficacy assessment indicators. These guidelines combined clinical symptoms and endoscopic examination as efficacy determination indicators, including remission conditions, clinical efficacy determination (remission, effectiveness, and ineffectiveness), and recurrence conditions. However, these criteria were not adopted in the included articles. In addition, for the selection of clinical observation indicators, most studies adopted clinical efficacy to compare effects; therefore, the subjectivity of effect assessment was stronger. Objective indicators, such as clinical symptom scoring or endoscopic and pathological scoring, were relatively rare; therefore, efficacy determination in these studies had a significant subjective tendency, and the reliability of the efficacy of acupuncture and moxibustion was reduced. In terms of long-term efficacy, most studies did not perform long-term follow-up. The studies that did perform follow-up did not describe the conditions of losses to follow-up (the number of patients lost to follow-up and the reasons for loss to follow-up) or the follow-up methods; therefore, the long-term efficacy of the acupuncture and moxibustion therapies used in these studies could not be determined.

## 5. Conclusion

In summary, the experimental design, methods, diagnosis, treatment, and efficacy assessment should be improved to provide high-quality evidence for clinical decision-making. With the methods and results of medical researches, the treatment effect of acupuncture and moxibustion on UC will be better observed in the future.

## Figures and Tables

**Table 1:** The use of diagnostic standards.

Serial number	Diagnostic standards	Number of articles
1	1978 Hangzhou conference on ulcerative diagnostic standards of the Chinese Society of Gastroenterology, Chinese Medical Association	4
2	1993 Taiyuan ulcerative colitis diagnostic criteria from the national conference on chronic noninfectious intestinal diseases	10
3	2000 Chengdu conference on diagnostic standards for inflammatory bowel disease of the Chinese Society of Gastroenterology, Chinese Medical Association	13
4	2003 Chongqing integrative diagnostic and treatment programs for ulcerative colitis of the professional committee on digestive system diseases of the Chinese Association of Integrative Medicine	2
5	2007 consensus on standardization of diagnosis and treatment of inflammatory bowel disease in China by the inflammatory bowel disease collaborative group of the Chinese Society of Gastroenterology, Chinese Medical Association	3
6	2009 consensus on diagnosis and treatment of ulcerative colitis using traditional Chinese medicine of the Digestive Diseases Branch of China Association of Traditional Chinese Medicine	2
7	2010 consensus on diagnosis and treatment of ulcerative colitis using traditional Chinese medicine of the Digestive Diseases Branch of China Association of Traditional Chinese Medicine	2
8	2010 practical guidelines for the diagnosis and treatment of inflammatory bowel disease of the World Gastroenterology Organization	1
9	2010 consensus on integrative diagnosis and treatment of ulcerative colitis of the professional committee on digestive system diseases of the Chinese Association of Integrative Medicine	2
10	Teaching materials, different types of books, and other sources	18
11	Self-formulated standards	10
12	No diagnostic standards	5

Note: A total of 9 articles adopted both traditional Chinese medicine and Western medicine standards. “Teaching materials, different types of books, and other sources” included 3 articles on “internal medicine,” 1 article on the industry standards of the State Administration of Traditional Chinese Medicine, 3 articles on “principles of guidelines for clinical studies of new drugs in traditional Chinese medicine,” 1 article on “basics of Western internal medicine,” 1 article on “diagnostic and treatment standards of symptoms and prescription selection in traditional Chinese medicine,” 3 articles on “practical internal medicine,” 1 article on “clinical diagnostic and treatment guidelines” of the Chinese Medical Association, 1 article on “diagnostics in traditional Chinese medicine,” 1 article on “complete modern acupuncture therapy,” 1 article on “basics of Western internal medicine,” 1 article on “diagnostic analysis and diagnosis of digestive diseases,” and 1 article of English literature.

**Table 2:** Statistical analysis of classification of intervention measures in experimental groups.

Serial number	Treatment methods	Frequency
1	*Acupuncture and moxibustion therapy combined with drug treatment *	Total of 29 articles (46%)
	Acupuncture and moxibustion + Western medicine (13 articles)	
	Acupuncture and moxibustion + traditional Chinese medicine (oral/enema) (12 articles)	
	Acupuncture and moxibustion + traditional Chinese medicine + Western medicine (4 articles)	
		
2	*Single or combination use of acupuncture and moxibustion therapy*	Total of 34 articles (54%)
	Acupuncture + moxibustion (17 articles)	
	Moxibustion (9 articles)	
	Balance cupping (1 article)	
	Acupoint application (2 articles)	
	Acupoint catgut embedding (2 articles)	
	Acupuncture + massage (tui na) (1 article)	
	Acupuncture + TDP (1 article)	
	Abdominal acupuncture + catgut embedding (1 article)	

**Table 3:** Statistical analysis of the use of moxibustion.

Serial number	Moxibustion methods	Number of articles
1	Moxa stick moxibustion	18
2	Ginger moxibustion	12
3	Moxibustion with medicinal cakes	7
4	Warm acupuncture	5
5	Thunder fire moxibustion	1
6	Direct moxibustion	1
7	Long snake moxibustion	1
8	Heat-sensitive moxibustion	1
9	Salt-separated moxibustion	1

**Table 4:** Statistical results of commonly used acupoints.

Acupoint	Frequency
Tianshu (ST25)	53
Zusanli (ST36)	49
Guanyuan (RN4)	42
Shangjuxu (ST37)	33
Zhongwan (RN12)	32
Dachangshu (BL25)	26
Pishu (BL20)	25
Qihai (RN6)	24
Shenshu (BL23)	19
Shenque (RN8)	17
Yinlingquan (SP9)	15
Sanyinjiao (SP6)	13

**Table 5:** Statistical results of commonly used meridians.

Meridians	Number of points
Yangming Stomach Channel of Foot	13
Taiyang Bladder Channel of Foot	11
Taiyin Spleen Channel of Foot	10
Ren Meridian	8
Kidney Channel of Foot-Shaoyin	5
Governor Meridian	4
Yangming Large Intestine Channel of Hand	4

**Table 6:** Statistical results of total number of treatment days.

Total number of treatment days	Number of articles
<10 days	1
10~19 days	11
20~29 days	7
30~39 days	14
40~49 days	7
50~59 days	1
60~69 days	6
70 days and above	1
Unknown	10

**Table 7:** Statistical results of single treatment duration.

Duration of a single treatment (e.g., retaining needles or moxibustion)	Number of articles
<20 min	11
20~30 min	30
>30 min	6
Not calculated or unknown^*∗*^	16

^*∗*^Not calculated: moxa Zhuangs are used to indirectly describe the moxibustion treatment time; unknown: a single treatment time is not clearly described.

**Table 8:** Utilization of efficacy assessment criteria.

Serial number	Efficacy assessment methods	Number of articles
1	1978 Hangzhou conference on ulcerative diagnostic standards of the Chinese Society of Gastroenterology, Chinese Medical Association	1
2	1992 standards for the diagnosis, symptom differentiation, and efficacy of chronic and nonspecific ulcerative colitis (CUC) (provisional programs) of the Professional Committee on digestive diseases of the Shanxi Linfen Symposium, Chinese Association of Integrative Medicine	1
3	1993 Taiyuan ulcerative colitis diagnostic criteria of the national conference on chronic noninfectious intestinal diseases	9
4	2000 Chengdu conference on diagnostic standards for inflammatory bowel disease of the Chinese Society of Gastroenterology, Chinese Medical Association	3
5	2007 consensus on standardization of diagnosis and treatment of inflammatory bowel disease in China by the inflammatory bowel disease collaborative group of the Chinese Society of Gastroenterology, Chinese Medical Association	3
6	2009 consensus on diagnosis and treatment of ulcerative colitis using traditional Chinese medicine of the Digestive Diseases Branch of the Chinese Medical Association	2
7	2010 consensus on diagnosis and treatment of ulcerative colitis using traditional Chinese medicine of the Digestive Diseases Branch of China Association of Traditional Chinese Medicine	1
8	*⟪*Principles of guidelines for clinical studies of new drugs in traditional Chinese medicine*⟫*	11
9	*⟪*Standards of efficacy and diagnosis of disease symptoms in traditional Chinese medicine*⟫*	3
10	90th National Integrative Medicine Symposium	1
11	Guidelines for diagnosis and treatment in integrative medicine (draft), Professional Committee on digestive diseases, Chinese Association of Integrative Medicine	1
12	English	1
13	Teaching materials, books, and other literature	7
14	None	2
15	Self-formulated	17

Note: the 7 articles with “teaching materials, books, and other literature” as efficacy assessment methods included 1 article on “chronic colitis,” 2 articles on “diagnosis of gastrointestinal diseases,” 1 article on “principles of guidelines for new drugs,” 1 article on “internal medicine,” and 2 articles on “sulfasalazine rectal suppository collaborative group: comparative study of the treatment of ulcerative colitis using sulfasalazine using rectal suppositories and oral tablets–analysis of 62 clinical cases.”
